# Non-contrast mDixon MR angiography of the neck: Comparison with time-of-flight MR angiography in normal subjects

**DOI:** 10.1097/MD.0000000000028351

**Published:** 2021-12-23

**Authors:** Tomohiro Mizoshiri, Morikatsu Yoshida, Seitaro Oda, Shota Tsumagari, Takeshi Nakaura, Kazunori Harada, Osamu Ikeda

**Affiliations:** a Department of Radiology, Amakusa Medical Center, 854-1 Jikiba, Kameba, Amakusa, Kumamoto, Japan; b Department of Diagnostic Radiology, Faculty of Life Sciences, Kumamoto University, 1-1-1 Honjo, Chuo-ku, Kumamoto, Japan; c Department of Surgery, Amakusa Medical Center, 854-1 Jikiba, Kameba, Amakusa, Kumamoto, Japan.

**Keywords:** image quality, magnetic resonance imaging, mDixon, neck MRA, time-of-flight

## Abstract

We investigated the feasibility of non-contrast three-dimensional modified Dixon (mDixon) magnetic resonance angiography (MRA) to evaluate the carotid artery.

We studied 30 normal patients who underwent non-contrast mDixon and conventional time-of-flight (TOF) MRA of the neck with a clinical 3T MR scanner. Carotid artery signal-to-noise ratio (SNR) and contrast-to-noise ratio were compared between mDixon-MRA and TOF-MRA. Two readers independently evaluated vessel sharpness, image contrast, and overall image quality using a 4-point scale.

SNR was significantly higher on mDixon-MRA than TOF-MRA (*P* < .01). There was no significant difference in contrast-to-noise ratio. The visual score for vessel sharpness was significantly higher on mDixon-MRA than TOF-MRA (*P* < .01), whereas the score for contrast was significantly higher on TOF-MRA (*P* < .01).

Although non-contrast three-dimensional mDixon-MRA showed lower visual contrast than conventional TOF-MRA, it provided images with significantly higher SNR and better vessel sharpness than TOF-MRA.

## 1 Introduction

Internal carotid artery (ICA) stenosis is an important cause of ischemic stroke, which may result in death or reduced quality of life.^[1]^ Therefore, its evaluation and management are clinically important. Although digital subtraction angiography (DSA) is the gold standard for evaluating ICA stenosis,^[2,3]^ it is an invasive procedure. Three-dimensional (3D) time-of-flight (TOF) magnetic resonance angiography (MRA) can provide high-quality arterial images and is currently widely used for non-invasive assessment of ICA stenosis.^[4]^ This also has the advantage of not requiring the use of contrast media. However, it is a flow-dependent sequence and blood flow turbulence often causes signal loss, resulting in blurred vessel visualization (reduced vessel sharpness) and stenosis overestimation.^[5,6]^

In non-contrast-enhanced flow-independent MRA, such as the Dixon-based sequence, intrinsic tissue parameters such as relaxation times and chemical shift are utilized to suppress background signals and generate relatively stable vessel contrast.^[7]^ The 2-point Dixon reconstruction method for decomposition of aqua/lipid, a variant of the in-phase/opposed-phase method, is traditionally used with clinical MR scanners. This method takes advantage of the intrinsic differences in the resonant frequency of fat and water protons to decompose their respective signals into separate images,^[8]^ producing a homogeneous fat and water separation that is less sensitive to B0 inhomogeneities.^[9]^ However, the Dixon method requires longer scan times and has lower scan parameter flexibility. The modified Dixon (mDixon) method overcomes these disadvantages^[10]^ and has proven to be clinically useful in various MRA applications^[11–14]^ but has not been fully evaluated in MRA of the neck. Based on its lower sensitivity to blood turbulence, we hypothesized that mDixon-based non-contrast MRA can provide a sharp and clear depiction of the carotid artery than conventional TOF-MRA. The purpose of this study was to investigate the feasibility of non-contrast 3D mDixon MRA to evaluate the carotid artery.

## 2 Materials and methods

### 2.1 Patient population

We prospectively enrolled 30 consecutive patients (18 female and 12 male; mean age ± standard deviation, 68.9 ± 14.5 years; age range, 27–89 years) with normal carotid ultrasonography findings who underwent non-contrast mDixon-MRA and conventional TOF-MRA between April 2019 and June 2019. This study was approved by the institutional review board of Amakusa Medical Center. Informed consent was obtained from all patients.

### 2.2 MRA sequence and parameters

All subjects underwent imaging on a 3T MR scanner (Ingenia; Philips Medical Systems, Amsterdam, Netherlands) with a 16-element phased-array Direct Digital RF receiver coil. After scout images were obtained, we performed conventional 3D TOF-MRA of the neck as a control followed by non-contrast 3D mDixon-MRA. The spatial resolution for 3D TOF-MRA was 0.5 × 0.79 × 1.1 mm and that for 3D mDixon-MRA was 1.2 × 1.19 × 1 mm; they differed in order to optimize their respective acquisition times. The scanning parameters for TOF-MRA were as follows: repetition time = 24 ms, echo time (TE) = 3.5 ms, flip angle = 20°, parallel imaging (SENSE = phase reduction 3, slice reduction 1), field of view = 200 × 150, matrix = 400 × 189, number of slices = 159, and acquisition time = 2 minutes 43 seconds. The mDixon-MRA parameters were: repetition time = 13 ms, TE = 1.43/2.6 ms, flip angle = 5°, parallel imaging (SENSE = phase reduction 1, slice reduction 1), field of view = 200 × 148, matrix = 168 × 123, number of slices = 150, and acquisition time = 1 minute 51 seconds (Table 1).

**Table 1:** Magnetic resonance imaging sequences and parameters.

	TOF-MRA	mDixon-MRA
Spatial resolution (mm)	0.5 × 0.79 × 1.1	1.2 × 1.19 × 1
TR (ms)	24	13
TE (ms)	3.5	1.43/2.6
Flip angle (°)	20	5
Matrix	400 × 189	168 × 123
Field of view (mm)	200 × 150	200 × 148
SENSE (phase × slice)	3 × 1	1 × 1
Number of slices	159	150
Acquisition time (min)	2:43	1:51

mDixon = modified Dixon, MRA = magnetic resonance angiography, TE = echo time, TOF = time-of-flight, TR = repetition time.

### 2.3 Quantitative image analysis

A board-certified radiologist with 6 years of MRA experience performed quantitative image analysis using the source images. Manually placed circular regions of interest (ROIs) were used to measure signal intensity (SI). Based on previous similar reports,^[15]^ we obtained SI of the common carotid artery (CCA), ICA origin, and mid-portion of the ICA (approximately 5 cm distal to the carotid bifurcation). ROIs were placed in the circumjacent air and sternocleidomastoid muscle to measure SI as a reference for image noise and the surrounding tissue, respectively. To minimize bias from single side measurements, we adopted the average of the left- and right-side values for each ROI site. The arterial signal-to-noise ratio (SNR) and contrast-to-noise ratio (CNR) between the arteries and perivascular tissue of each MRA method were calculated using the following formulas:


$$\begin{array}{lll} \text{Contrast} & = & \text{S}{\text{I}}_{\text{artery}}\text{-S}{\text{I}}_{\text{tissue}}\\ \text{SNR} & = & \text{S}{\text{I}}_{\text{artery}}/\text{S}{\text{D}}_{\text{noise}}\\ \text{CNR} & = & \text{Contrast}/\text{S}{\text{D}}_{\text{noise}} \end{array}$$

### 2.4 Qualitative image analysis

To evaluate image quality of the different sequences, we performed qualitative image analysis on a PACS viewer (SYNAPSE; Fujifilm Corp., Tokyo, Japan). Available images included axial source images and maximum intensity projection images. Images acquired with the 2 MRA methods were randomized. Two board-certified radiologists with 6 and 14 years of MRI experience, respectively, who were blinded to the acquisition parameters and techniques, independently graded image contrast, vessel sharpness (apparent flow-related dephasing), and overall image quality using a 4-point subjective scale: image contrast and overall image quality (1 = unacceptable, 2 = poorer than average, 3 = good, 4 = excellent), image sharpness (1 = blurry, 2 = poorer than average, 3 = better than average, 4 = sharpest). Inter-observer disagreement was settled by consensus. For qualitative analysis, a total of 60 carotid arteries were evaluated (30 patients, left and right). The radiologists were able to adjust window level and width during the qualitative assessment. The number of arteries with inappropriate image quality (score = 1 or 2) was recorded for each assessment parameter.

### 2.5 Statistical analysis

Statistical analyses were performed using JMP statistical software version 12.0 (SAS Institute, Inc., Cary, NC, USA). All numerical values are reported as means ± standard deviation. SNR, CNR, and qualitative scores were compared between mDixon-MRA and TOF-MRA using the paired *t* test or Wilcoxon signed-rank test as appropriate. The number of arteries with inappropriate image quality was compared using the Fisher exact test. *P* < .05 was considered significant.

## 3 Results

### 3.1 Quantitative analysis

All neck MRA studies were successfully completed. As shown in Table 2, the SNR of the CCA, ICA origin, and mid-portion of the ICA were significantly higher on mDixon-MRA than TOF-MRA (*P* < .01, *P* < .01, and *P* < .01, respectively). There was no significant difference between the 2 methods in CNR (CCA, *P* = .05; ICA origin, *P* = .52; mid-portion of the ICA, *P* = .52).

**Table 2:** Quantitative analysis comparing mDixon-MRA and TOF-MRA.

	Signal-to-noise ratio		
	TOF-MRA	mDixon-MRA	*P* value
CCA	58.3 ± 17.9	66.3 ± 20.3	<.01
ICA origin	57.0 ± 17.4	67.0 ± 20.8	<.01
Mid-portion of ICA	58.5 ± 18.0	68.1 ± 21.9	<.01

	Contrast noise ratio		
	TOF-MRA	mDixon-MRA	*P* value
CCA	44.1 ± 15.1	41.7 ± 14.2	.05
ICA origin	42.8 ± 14.7	42.4 ± 14.6	.52
Mid-portion of ICA	42.8 ± 14.7	42.4 ± 14.6	.52

Values are mean ± standard deviation. *P* < .05 was considered significant. CCA = common carotid artery, ICA = internal carotid artery, mDixon = modified Dixon, MRA = magnetic resonance angiography, TOF = time-of-flight.

### 3.2 Qualitative analysis

The results of our qualitative image quality assessment are shown in Table 3. The visual score for vessel sharpness was significantly higher on mDixon-MRA than TOF-MRA (*P* < .01), whereas the score for contrast was significantly higher on TOF-MRA than mDixon-MRA (*P* < .01). There was no significant difference between the 2 methods in overall image quality (*P* = .40).

**Table 3:** Qualitative analysis comparing mDixon-MRA and TOF-MRA.

	TOF-MRA	mDixon-MRA	*P* value
Image contrast (both sides)	3.6 ± 0.4	2.9 ± 0.5	<.01
Left-side	3.5 ± 0.4	3.0 ± 0.4	<.01
Right-side	3.6 ± 0.4	3.1 ± 0.3	<.01
Vessel sharpness (both sides)	3.2 ± 0.6	3.8 ± 0.4	<.01
Left-side	3.2 ± 0.6	3.7 ± 0.4	<.01
Right-side	3.4 ± 0.6	3.8 ± 0.2	<.01
Overall image quality (both sides)	3.5 ± 0.3	3.5 ± 0.2	.40
Left-side	3.6 ± 0.3	3.5 ± 0.3	.11
Right-side	3.7 ± 0.3	3.5 ± 0.2	.07

Values are mean ± standard deviation. *P* < .05 was considered significant. mDixon = modified Dixon, MRA = magnetic resonance angiography, TOF = time-of-flight.

**Figure 1:**
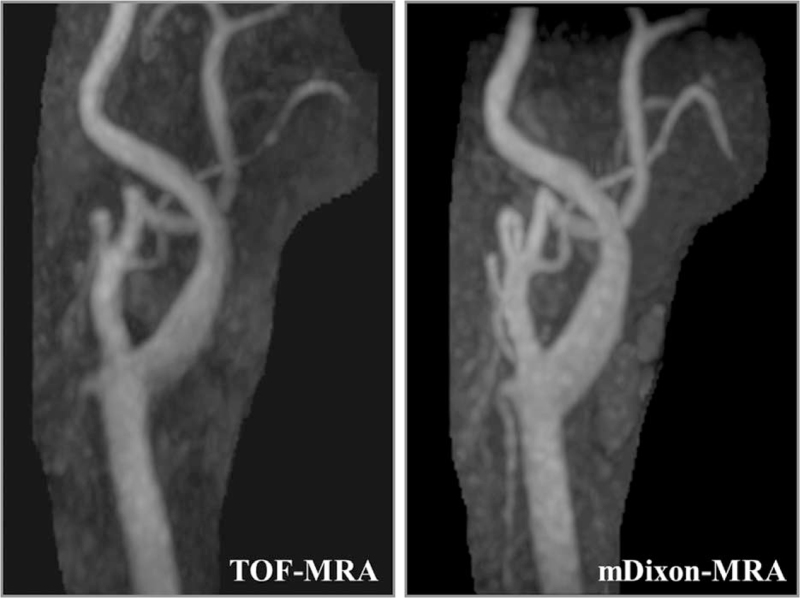
Maximum intensity projections of the carotid bifurcation in a 64-year-old man who underwent MRA of the neck. The visual scores for image contrast, vessel sharpness, and overall image quality were 4, 3, and 4, respectively, for TOF-MRA, and 4, 4, and 4, respectively, for mDixon-MRA. mDixon = modified Dixon, MRA = magnetic resonance angiography, TOF = time-of-flight.

**Figure 2:**
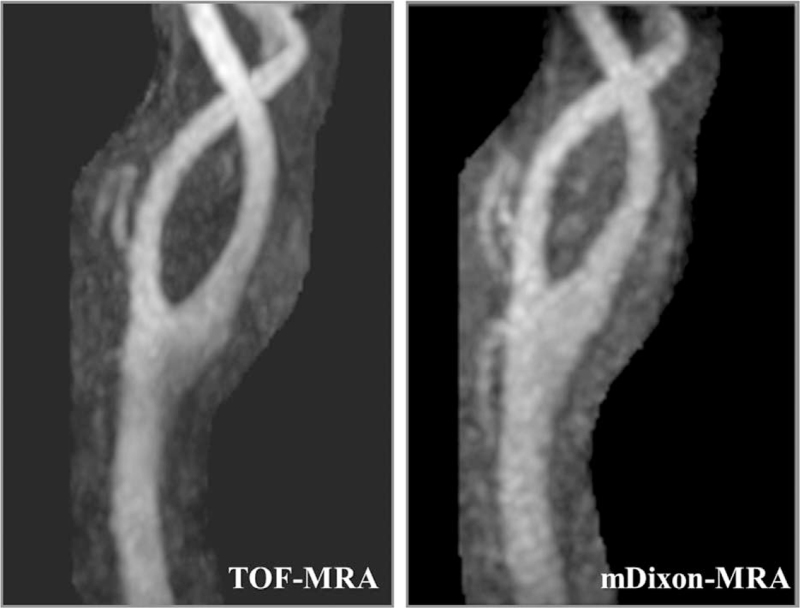
Maximum intensity projections of the carotid artery bifurcation in a 66-year-old man who underwent MRA of the neck. The visual scores for image contrast, vessel sharpness, and overall image quality were 4, 3, and 4, respectively, for TOF-MRA, and 3, 4, and 3, respectively, for mDixon-MRA. mDixon = modified Dixon, MRA = magnetic resonance angiography, TOF = time-of-flight.

Of the 60 arteries evaluated, 9 were judged as inappropriate image quality (score = 1 or 2) for image contrast, 2 for vessel sharpness, and 2 for overall image quality on mDixon-MRA. On TOF-MRA, the number of arteries judged as inappropriate image quality was 0 for image contrast, 11 for vessel sharpness, and 2 for overall image quality. The difference was significant for image contrast and vessel sharpness (*P* < .01) but not overall image quality (Table 4). Figures 1 and 2 show 2 representative cases.

**Table 4:** Number of arteries judged as inappropriate image quality (score = 1 or 2).

	TOF-MRA	mDixon-MRA	*P* value
Image contrast	0	9	<.01
Vessel sharpness	11	2	<.01
Overall image quality	2	2	.69

*P* < .05 was considered significant. mDixon = modified Dixon, MRA = magnetic resonance angiography, TOF = time-of-flight.

## 4 Discussion

Our results demonstrate that non-contrast neck MRA using the mDixon method provides images with higher SNR and better vessel sharpness than conventional TOF-MRA. Moreover, the acquisition time was shorter for mDixon-based MRA than TOF-MRA. This suggests that mDixon-MRA can be less sensitive to blood turbulence, enabling better carotid artery depiction than conventional TOF-MRA. Although further studies should assess the applicability of our findings, mDixon-MRA may assess the ICA more accurately compared to TOF-MRA.

Vessel sharpness was significantly better with 3D mDixon-MRA than 3D TOF-MRA. This could be due to flow void artifact related to blood flow turbulence seen on 3D TOF-MRA,^[5]^ which often leads to stenosis overestimation. Flow void artifact can be related to both TE and spatial resolution.^[6]^ However, in our study, 11 of 60 arteries showed lower vessel sharpness, even though we used a relatively short TE of 3.5 ms in 3D TOF-MRA. Weber et al^[4]^ acquired 3D TOF-MRA images using almost identical TE and spatial resolution parameters as our study and reported that it overestimated proximal ICA stenosis. On the mDixon sequence, water and fat images are based on differences in the resonant frequency of fat and water protons.^[8]^ The generated set of water images are characterized by robust homogeneous fat suppression and significantly less artifact.^[16]^ Moreover, the mDixon sequence is relatively insensitive to B0 and B1 field inhomogeneities.^[10]^ On the other hand, although the mDixon sequence can be acquired with shorter TE than the TOF sequence, the spatial resolution was lower on mDixon-MRA than TOF-MRA in this study. Therefore, factors other than TE and spatial resolution may also be related to flow void artifact.

We found that the visual image contrast was significantly lower on mDixon-MRA than TOF-MRA. However, mDixon-MRA SNR and CNR were not inferior. This may be due to the lower image noise of mDixon-MRA, which can be explained by several factors. First, the pixel size was larger on mDixon-MRA than TOF-MRA. Second, mDixon-MRA can acquire water images, which are characterized by robust homogeneous fat suppression. Third, mDixon-MRA is relatively insensitive to B0 and B1 field inhomogeneities. The mDixon-MRA water imaging shows the resonant frequency of water protons, thus background SI is not suppressed, as in TOF-MRA. In previous reports, T2-prep plus and non-selective inversion recovery have been applied with a short inversion time to suppress background tissue signal.^[12]^ These techniques can potentially increase contrast and improve the image quality of mDixon-based neck MRA.

This study has several limitations. First, we investigated a small number of normal subjects at a single center, thus selection bias may have been introduced. Future large-scale clinical studies involving normal and pathological vessels are needed to validate our results. Second, we did not evaluate the diagnostic performance of mDixon-MRA for detecting ICA stenosis by correlating our imaging findings with DSA results because DSA imaging was not available in most study patients. Rather, we focused on comparing the quality of images of neck MRA obtained with mDixon with those obtained with TOF. Diagnostic performance of mDixon-MRA should be compared to that of TOF-MRA using the patients with ICA stenosis in the future. Third, it is unclear whether the MRA scan parameters of the 2 methods in our study were optimal for neck MRA. The MRA imaging parameters may need further optimization.

In conclusion, although non-contrast 3D mDixon-MRA showed lower visual contrast than conventional 3D TOF-MRA, it provided images with significantly higher SNR and better vessel sharpness than TOF-MRA in normal subjects. However, further research is needed to confirm the findings.

## Author contributions

**Conceptualization:** Seitaro Oda.

**Data curation:** Tomohiro Mizoshiri.

**Formal analysis:** Seitaro Oda, Shota Tsumagari.

**Investigation:** Morikatsu Yoshida, Seitaro Oda.

**Methodology:** Morikatsu Yoshida, Seitaro Oda.

**Supervision:** Takeshi Nakaura, Kazunori Harada.

**Writing – original draft:** Tomohiro Mizoshiri.

**Writing – review & editing:** Seitaro Oda, Osamu Ikeda.

## References

[1] Ntaios G Perlepe K Sirimarco G. Carotid plaques and detection of atrial fibrillation in embolic stroke of undetermined source. Neurology 2019;92:e2644–52. 31068479 10.1212/WNL.0000000000007611

[2] Jaff MR. Imaging the carotid bifurcation: toward standardization. Semin Vasc Surg 2008;21:73–9. 18565413 10.1053/j.semvascsurg.2008.03.001

[3] Anzalone N Scomazzoni F Castellano R. Carotid artery stenosis: intraindividual correlations of 3D time-of-flight MR angiography, contrast-enhanced MR angiography, conventional DSA, and rotational angiography for detection and grading. Radiology 2005;236:204–13. 15955853 10.1148/radiol.2361032048

[4] Weber J Veith P Jung B. MR angiography at 3 Tesla to assess proximal internal carotid artery stenoses: contrast-enhanced or 3D time-of-flight MR angiography? Clin Neuroradiol 2015;25:41–8. 24384680 10.1007/s00062-013-0279-x

[5] Nederkoorn PJ van der Graaf Y Eikelboom BC van der Lugt A Bartels LW Mali WP. Time-of-flight MR angiography of carotid artery stenosis: does a flow void represent severe stenosis? AJNR Am J Neuroradiol 2002;23:1779–84. 12427639 PMC8185836

[6] Lev MH Romero JM Gonzalez RG. Flow voids in time-of-flight MR angiography of carotid artery stenosis? It depends on the TE!. AJNR Am J Neuroradiol 2003;24:2120. PMC8148931 14625247

[7] Wheaton AJ Miyazaki M. Non-contrast enhanced MR angiography: physical principles. J Magn Reson Imaging 2012;36:286–304. 22807222 10.1002/jmri.23641

[8] Dixon WT. Simple proton spectroscopic imaging. Radiology 1984;153:189–94. 6089263 10.1148/radiology.153.1.6089263

[9] Ma J. Breath-hold water and fat imaging using a dual-echo two-point Dixon technique with an efficient and robust phase-correction algorithm. Magn Reson Med 2004;52:415–9. 15282827 10.1002/mrm.20146

[10] Eggers H Brendel B Duijndam A Herigault G. Dual-echo Dixon imaging with flexible choice of echo times. Magn Reson Med 2011;65:96–107. 20860006 10.1002/mrm.22578

[11] Homsi R Gieseke J Kukuk GM. Dixon-based fat-free MR-angiography compared to first pass and steady-state high-resolution MR-angiography using a blood pool contrast agent. Magn Reson Imaging 2015;33:1035–42. 26220860 10.1016/j.mri.2015.07.005

[12] Yoneyama M Zhang S Hu HH. Free-breathing non-contrast-enhanced flow-independent MR angiography using magnetization-prepared 3D non-balanced dual-echo Dixon method: a feasibility study at 3 Tesla. Magn Reson Imaging 2019;63:137–46. 31425807 10.1016/j.mri.2019.08.017

[13] Stafford RB Sabati M Haakstad MJ Mahallati H Frayne R. Unenhanced MR angiography of the renal arteries with balanced steady-state free precession Dixon method. AJR Am J Roentgenol 2008;191:243–6. 18562753 10.2214/AJR.07.3076

[14] Kourtidou S Jones MR Moore RA. mDixon ECG-gated 3-dimensional cardiovascular magnetic resonance angiography in patients with congenital cardiovascular disease. J Cardiovasc Magn Reson 2019;21:52. 31391061 10.1186/s12968-019-0554-3 PMC6686451

[15] Hoelter P Lang S Weibart M. Prospective intraindividual comparison of gadoterate and gadobutrol for cervical and intracranial contrast-enhanced magnetic resonance angiography. Neuroradiology 2017;59:1233–9. 28913611 10.1007/s00234-017-1922-z

[16] Riederer SJ Stinson EG Weavers PT. Technical aspects of contrast-enhanced MR angiography: current status and new applications. Magn Reson Med Sci 2018;17:03–12. 10.2463/mrms.rev.2017-0053 PMC5760227 28855470

